# Optimizing Microfluidic Channel Design with Tilted Rectangular Baffles for Enhanced mRNA-Lipid Nanoparticle Preparation

**DOI:** 10.1021/acsbiomaterials.4c02373

**Published:** 2025-05-21

**Authors:** Mingzhi Yu, Dongsheng Liu, Pranay Shah, Bei Qiu, Allen Mathew, Liang Yao, Tianyu Guan, Hengji Cong, Nan Zhang

**Affiliations:** † Centre of Micro/Nano Manufacturing Technology (MNMT-Dublin), School of Mechanical & Materials Engineering, 8797 University College Dublin, Dublin 4 D04 V1W8, Ireland; ‡ Department of Aerospace and Mechanical Engineering, South East Technological University, Carlow R93 V960, Ireland; § The Centre for Research and Enterprise in Engineering (engCORE), South East Technological University, Carlow R93 V960, Ireland; ∥ Charles Institute of Dermatology, School of Medicine, University College Dublin, Dublin D04 V1W8, Ireland

**Keywords:** microfluidic, CFD simulations, lipid nanoparticles, transfection

## Abstract

RNA therapeutics represent a pivotal advancement in contemporary medicine, pioneering innovative treatments in oncology and vaccine production. The inherent instability of RNA and its delivery challenges necessitate the use of lipid-based nanoparticles as crucial transport vehicles. This research focuses on the design, simulation, and optimization of various microfluidic channel configurations for fabricating poly­(dimethylsiloxane) (PDMS) microfluidic chips, aimed at producing lipid nanoparticles (LNPs) encapsulating green fluorescent protein mRNA (GFP mRNA). Aiming for high mixing efficiency and acceptable pressure drop suitable for scale-up, we designed and improved multiple microfluidic channels featuring flow focusing and diverse tilted rectangular baffle structures via computational fluid dynamics (CFD). Simulation results indicated that baffle angles ranging from 70 to 90° exhibited similar mixing efficiencies at different total flow rates, with pressure drops increasing alongside the baffle angle. Additionally, increasing the baffle length at a fixed angle of 70° not only improved mixing efficiency but also increased the pressure drop. To validate these findings, PDMS microfluidic chips were fabricated for all designs to prepare empty LNPs. The baffle structure with a 70° angle and 150 μm length was identified as the best configuration based on both simulation and experimental results. This optimal design was then used to prepare LNPs with varying GFP mRNA concentrations, demonstrating that an N/P ratio of 5.6 yielded the highest transfection efficiency from *in vitro* experiments. This work not only advances the production of lipid-based nanoparticles through microfluidics but also provides a scalable and reproducible method that can potentially enhance the clinical translation of RNA therapeutics.

## Graphical abstract

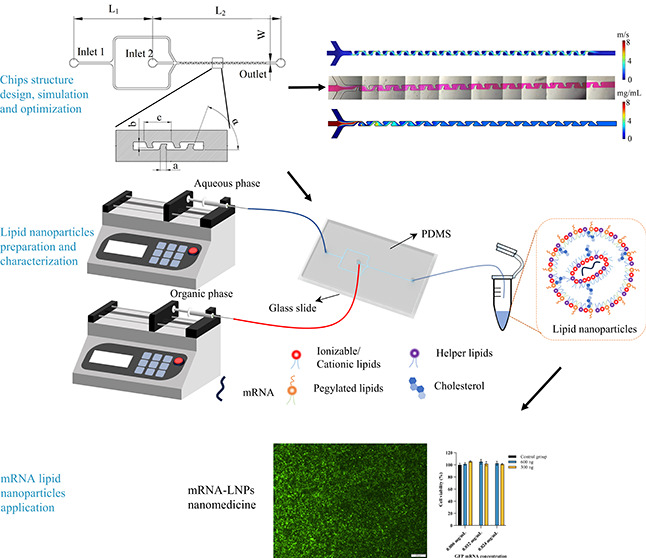

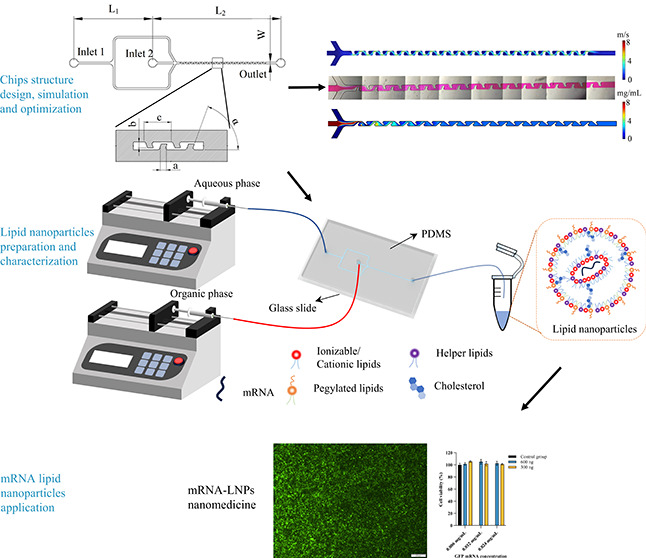

## 1 Introduction

RNA therapeutics hold immense potential in clinical applications, such as cancer therapy and vaccine development.^1,2^ Carriers like lipid-based nanoparticles, polymeric nanoparticles, and inorganic nanoparticles are essential for protecting and delivering RNA into cells.^3,4^ Lipid-based nanoparticles are becoming increasingly prominent due to the success of mRNA (mRNA) lipid nanoparticle vaccines, such as the BNT162b2 vaccine from Pfizer and BioNTech and the mRNA-1273 vaccine from Moderna.^5^ These vaccines have saved millions of lives during the COVID-19 pandemic.^6^

Generally, lipids are dissolved in an organic phase solution (usually ethanol), while the payload (RNA/DNA) is dissolved in an aqueous phase solution.^7^ Several factors can affect the properties of lipid nanoparticles (LNPs), including the composition of lipids (cationic/ionizable lipids, phospholipids, cholesterol, and PEGylated lipids), lipid ratios, lipid concentrations, and the concentrations of RNA/DNA, as well as the N/P ratio.^8–12^ N/P ratio refers to the molar ratio of the amine group within the cationic lipid (N) to phosphate groups (P) from RNA/DNA. Therefore, there exist thousands of formulations for the lipid nanoparticles, necessitating to find the optimal formulation to achieve the highest transfection efficiency. On the other hand, the characteristics of lipid nanoparticles will also influence their transfection efficiency, including the size, polydispersity index (PDI), and zeta potential perspectives.^13–16^ For example, small nanoparticles (<5 nm) are filtered and removed from the vascular compartment by the kidneys.^17,18^ Larger nanoparticles (10–150 nm) exhibit effective drug encapsulation efficiency and longer circulation times, leading to better treatment outcomes.^18–20^ However, a bigger nanoparticle size is not always better. Larger nanoparticles struggle to pass through healthy organs,^21^ and the maximum size for treating tumor interstitium is 400 nm.^19,22^ Zeta potential, or surface charge, is also crucial as it influences nanoparticle distribution and intracellular endocytosis rates.^15,23,24^ Therefore, LNPs with a diameter less than 150 nm, a PDI less than 0.2, and low zeta potential are more conducive to RNA delivery.

The traditional method for preparing LNPs involves mixing aqueous and organic phase solutions in a 1:1 volume ratio using pipet or vortex mixing.^25,26^ While these methods are easy and cost-effective, they suffer from poor reproducibility and controllability.^25^ An alternative approach is microfluidic mixing, which is a multidisciplinary technology combining engineering, physics, and biotechnology.^27–31^ Microfluidic chips offer controllable production with high reproducibility and adjustable ratios between aqueous and organic phase solutions.^30,32^ Additionally, laminar flow within microfluidic chips enhances diffusion rates, allowing rapid optimization of lipid nanoparticle production.^33,34^

There are several microfluidic designs for preparing lipid-based nanoparticles in the formulations. Y-junction, T-junction, and flow focusing microfluidic structures allow rapid mixing of aqueous and organic phase solutions, leading to high mixing efficiency, thus reducing size and PDI.^35–39^ For instance, Hood et al.^38^ successfully used a flow focusing microfluidic device to synthesize liposomes around 80 nm in size with a PDI lower than 0.2, demonstrating good stability. Additionally, staggered herringbone microfluidic mixing (SHM) structures have been designed and used to synthesize LNPs.^40,41^ Sato et al.^41^ employed SHM devices, which consist of two-layer channels (mixer structures and main flow channels), to prepare siRNA-loaded LNPs with sizes ranging from 32 to 67 nm. Furthermore, Kimura et al.^42^ designed devices with specific baffle structures to synthesize controllable LNPs. To the best of the authors’ knowledge, although some research has been conducted on baffle structure design, limited studies have focused on how baffle characteristics, such as length and angle, affect mixing and subsequent LNP synthesis. Additionally, microfluidic channel designs must address pressure drop reduction to facilitate seamless transition from low-volume formulation to large-volume scale-up production.^43,44^

In this study, microfluidic channels with different tilted rectangular baffle structures were designed, simulated, and optimized their performance in terms of mixing efficiency and pressure drop. The baffle structure can accelerate the mixing of two liquids by altering the flow velocity and direction of the solutions. Poly­(dimethylsiloxane) (PDMS) microfluidic chips with these structures were manufactured and used to prepare LNPs at different total flow rates. We observed that nanoparticle size decreased with increasing total flow rate, and dilution also reduced size and PDI. Finally, the microfluidic chip with high mixing performance, according to the simulation and empty LNPs, results was used to carry green fluorescent protein (GFP) mRNA to test transfection efficiency.

## 2 Materials and Methods

### 2.1 Materials

Acetone, 2-propanol (IPA), trichloro­(1*H*,1*H*,2*H*,2*H*-perfluorooctyl) silane, tris buffer, ethanol, and cholesterol were procured from Sigma-Aldrich (Darmstadt, Germany). The ultrasonic cleaner was acquired from GT Sonic (Guangdong, China). The PDMS surface modification was performed using oxygen plasma with a plasma cleaner (ZEPTO-W6, Diener electronic, Baden-Württemberg, Germany). A spin coater from Laurell Technologies (North Wales) was employed to spread the adhesive voucher. SU8 2050 and SU8 Developer were obtained from Kayaku Advanced Materials (Massachusetts). The UV-LED masking system, UV-KUB 2, was sourced from KLOE (France). The SYLGARD 184 Silicone Elastomer Kit (PDMS) was supplied by Ellsworth Adhesives (Ireland). Microscope equipment was provided by Brunel Microscopes (U.K.). The syringe pump was obtained from KD Scientific, Inc. The Litesizer 500, used to measure nanoparticle size, PDI, and zeta potential, was obtained from Anton Paar (Austria). Dimethyldioctadecylammonium (18:0 DDAB), 1,2-distearoyl-*sn*-glycero-3-phosphocholine (18:0 DSPC), and 1,2-dimyristoyl-rac-glycero-3-methoxypolyethylene glycol-2000 (DMG-PEG 2000) were purchased from Avanti Polar Lipids, Inc. (Alabama). Phosphate buffered saline tablets were sourced from Fisher Scientific (Pittsburgh). GFP mRNA was ordered from GenScript Biotech. DMEM 6249 was purchased from Merck Life Science. Fetal bovine serum (FBS), alamarBlue Cell Viability Reagent, and penicillin/streptomycin were ordered from Thermo Fisher Scientific (Waltham, MA).

### 2.2 Microfluidic Channels Design and Simulations

Flow focusing was designed to allow the meeting of two-phase solutions, while straight mixing channels with tilted rectangular baffle structures were designed to facilitate their mixing, as shown in Figure 1a,1b. The distance between inlet 1 and inlet 2 (*L*_1_) was 9 mm, and the distance between inlet 2 and the outlet (*L*_2_) was 15 mm. The channel width (*w*) was 200 μm, the baffle width (*a*) was 100 μm, and the distance between two adjacent baffle structures (*c*) was 300 μm. The baffle angle (α) and length (*b*) were considered for their impact on the mixing efficiency and pressure drop. Baffle angles ranging from 40 to 90° were initially designed and simulated. Based on the simulation results, the optimal baffle angle was selected to test the effect of different baffle lengths (100, 125, 150, and 175 μm) on mixing efficiency and pressure drop. The mixing channel consists of 15 baffle cycles with each cycle defined as a repeating unit composed of alternating baffle structures. Two representative cycles are shown in Figure 1b. The detailed parameter settings for channel design are presented in Tables 1 and 2.

**Figure 1:**
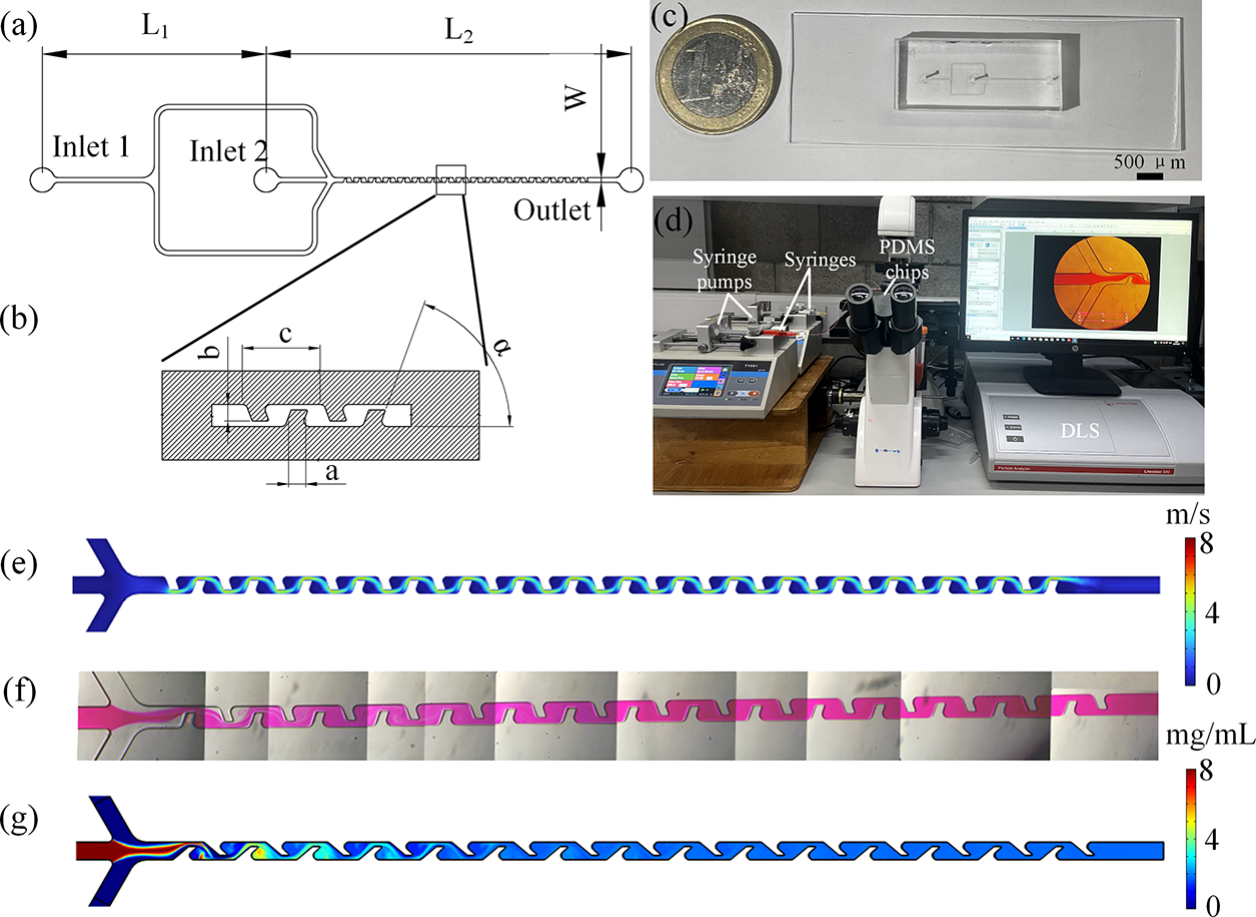
Baffle structure microfluidic design and mixing results: (a) Geometry of the microfluidic channels design. (b) Detailed design of the mixing channel unit. (c) PDMS microfluidic chip. (d) Experiment setup. (e) Velocity streamline plot simulation result of baffle structure with a 70° angle and 150 μm length at 1200 μL/min. (f) Mixing results using PDMS microfluidic chip with 70° angle and 150 μm length using rhodamine B at 1200 μL/min. (g) Concentration simulation results of baffle structure with a 70° angle and 150 μm length at 1200 μL/min.

**Table 1:** Baffle Structure Parameters with Different Angles

parameter				
chip no.	*a* (μm)	*b* (μm)	*c* (μm)	α (°)
1	100	150	300	40
2	100	150	300	50
3	100	150	300	60
4	100	150	300	70
5	100	150	300	80
6	100	150	300	90

**Table 2:** Baffle Structure Parameters with Different Lengths at a Fixed Angle α = 70°

parameter			
chip no.	*a* (μm)	*b* (μm)	*c* (μm)
7	100	100	300
8	100	125	300
4	100	150	300
9	100	175	300

Computational fluid dynamics (CFD) simulation was employed to analyze how baffle structures influence the mixing efficiency and pressure drop. Three-dimensional models were created based on the aforementioned specifications and imported into COMSOL Multiphysics 6.1 (COMSOL, Inc., Burlington, MA). Water was used as the working fluid. The density and dynamic viscosity were set as 997 kg/m^3^ and 8.9 × 10^–4^ kg/(m·s) at 25 °C, respectively.^45^ The diffusion coefficient of rhodamine B (RB) was 3.2 × 10^–10^ m^2^/s.^46^ The concentration of RB at inlet 1 was set to 0 mg/mL, and at inlet 2, it was set to 8 mg/mL, allowing for the evaluation of diffusion between the two inlet solutions. The total flow rates were set at 300, 600, 900, and 1200 μL/min. Based on previous studies, the optimal ratio between the aqueous phase and the organic phase for preparing LNPs was found to be 3:1; hence, the flow rate ratio between inlet 1 and inlet 2 was maintained at 3:1.^47,48^ To evaluate the spatial evolution of mixing, cross-sectional planes perpendicular to the flow direction were selected at various positions along the mixing channel for the observation and quantification of the mixing performance. The mixing efficiency, denoted by the mixing index, which indicates the uniformity of mixing, was calculated in MATLAB according to the following equation

1 $$mixing\;index=\left(1-\frac{1}{\mathrm{c̅}}\sqrt{\frac{\sum_{1}^{n}{\left(c_{i}-\mathrm{c̅}\right)}^{2}}{n}}\right)\times 100\%$$

where *c*_*i*_ denotes the concentration value at each pixel of concentration image, *c̅* is the average concentration fraction across the concentration image, and *n* denotes the number of pixels.^49,50^

### 2.3 PDMS Microfluidic Devices Manufacturing

At room temperature, a 4 in. silicon wafer was sequentially ultrasonically cleaned in acetone, isopropanol, and deionized water for 10 min each. After drying with air gas, the wafer was baked on a hot plate at 120 °C for 5 min to remove any residual moisture. After cleaning, the 4 in. silicon wafer was placed on a spin coater and evenly coated with a 100 μm thick layer of SU8 2050. The wafer was then transferred to a hot plate for soft baking. Following this, the wafer was exposed to UV light using a UV-KUB 2 system. After the postbake process, the unexposed photoresist was removed from the silicon wafer using SU8 developer, leaving the desired channel pattern. Finally, the PDMS replicated the pattern and was bonded onto the slide for testing. A PDMS microfluidic chip is shown in Figure 1c. The connection between the syringe and the PDMS chip was achieved by directly inserting tubing into prepunched inlet holes. These holes were slightly smaller than the outer diameter of the tubing, creating a tight press-fit seal that ensured a secure and leak-free connection during the operation.

### 2.4 Empty LNPs Preparation Using Microfluidic Chips

The experimental setup is shown in Figure 1d. Lipids DDAB, cholesterol, DSPC, and DMG-PEG2000 were dissolved in absolute ethanol at a weight ratio of 40:48:10:2, achieving a concentration of 8 mg/mL. One syringe filled with Tris buffer was placed on a syringe pump and connected to inlet 1. Another syringe containing the lipids in ethanol was placed on a different syringe pump and connected to inlet 2. The flow rates of the two syringe pumps were set to maintain a Tris buffer to lipid solution ratio of 3:1, with total flow rates of 300, 600, 900, and 1200 μL/min. After discarding the initial 200 μL of waste, 1 mL of LNPs was collected from the outlet. The sample was then diluted 5-fold with Tris buffer or PBS, and the size and PDI of the LNPs were measured using a Litesizer 500.

### 2.5 GFP mRNA LNPs Preparation and Characterization

Based on the simulation results and the measured sizes of the LNPs, the optimal baffle structure in the microfluidic chip was selected for testing with drug-loaded nanoparticles. GFP mRNA was dissolved in Tris buffer at concentrations of 0.04, 0.08, and 0.16 mg/mL. The syringe containing GFP mRNA in Tris buffer replaced the previous syringe with Tris buffer and was injected to mix with the lipids in ethanol at a total flow rate of 1200 μL/min. To ensure stable flow conditions, 200 μL of waste solution was first collected, followed by the collection of 500 μL of the LNP formulation for downstream analysis. The GFP mRNA LNPs were collected from the outlet and diluted five times with PBS buffer. After dilution, the final GFP mRNA concentration was 0.006, 0.012, and 0.024 mg/mL, and the final lipid concentration was 0.4 mg/mL. Also, the N/P ratios for 0.006, 0.012, and 0.024 mg/mL mRNA were 22.05, 11.253, and 5.626, respectively. The size, PDI, and zeta potential of the LNPs were measured using a Litesizer 500. Encapsulation efficiency was determined using the RiboGreen RNA Assay Kit and calculated using the following equation

2 $$e\mathrm{ncapsulation efficiency}\left(\%\right)=\frac{t\mathrm{otal mRNA concentration}-m\mathrm{easured mRNA concentration}}{\mathrm{to}\mathrm{tal mRNA concentration}}\times 100\%$$

The GFP mRNA LNPs were then ultrafiltered using an Amicon Ultra Centrifugal Filter (10 kDa MWCO) to prepare them for the cell experiment.

### 2.6 Cell Culture and Transfection

Human embryonic kidney (HEK) cells were cultured in DMEM supplemented with 10% FBS and 1% penicillin/streptomycin. Subsequently, 25,000 HEK cells per well were seeded in a 96-well plate. After 24 h of incubation at 37 °C with 5% CO_2_ in a humidified incubator, the medium was replaced with a mixture of GFP mRNA LNPs and fresh culture medium, bringing the total volume to 200 μL per well. Specifically, 50 or 25 μL of GFP mRNA LNPs at concentrations of 0.006, 0.012, or 0.024 mg/mL were added, corresponding to a final mRNA dose ranging from 150 ng to 1.2 μg per well, depending on the concentration and volume used. Following 48 h of further incubation under the same conditions, an Olympus IX81 fluorescence microscope (Olympus, Tokyo, Japan) was used to visualize the expression of the reporter GFP. The intensity was analyzed and semiquantified using ImageJ software (NIH, Bethesda, MD). Additionally, cell viability was assessed using the alamarBlue Cell Viability Reagent. A SpectraMax M3 multiplate reader (Molecular Devices, San Jose, CA) was used for excitation/emission values determination, which were recorded at 570 and 590 nm after incubation for 1.5 h protected from light at 37 °C.

### 2.7 Statistical Analysis

The mean ± standard deviation (±SD) was presented for all data. One-way ANOVA with Dunnett’s multiple-comparison tests was used to analyze the difference for different groups using Prism 8.0 (GraphPad). A difference was considered significant if *P* < 0.05 (*P < 0.05, **P < 0.01, ***P < 0.001, ****P < 0.0001).

## 3 Results

### 3.1 Simulation Results

To assess the effects of tilted rectangular baffle structures on the mixing efficiency and pressure drop, we utilized CFD simulations. The simulation included a velocity streamline plot of a baffle structure with a 70° angle and 150 μm length, under a flow rate of 1200 μL/min, as shown in Figure 1e. This showed an increased flow speed through the narrowed channel sections, enhancing the mixing of two-phase solutions. Both experimental and simulation results for the mixing index, presented in Figure 1f,g, demonstrated comparable outcomes. Initially, distinct colors were observed due to inadequate mixing. However, a uniform color emerged after the solution was passed through multiple baffle structures. CFD simulation results of concentration profiles from top and cross-sectional planes are shown in Figures S1 and S3, while mixing index results are shown in Figure 2a–d,g–j. The labels “1st”, “2nd”,..., and “15th” refer to the positions of repeating baffle cycles along the microfluidic mixing channel. Pressure drop simulations at different total flow rates, baffle angles, and baffle length profiles from top are shown in Figures S2 and S4, and the corresponding inlet–outlet pressure differences are displayed in Figure 2e,f,k,l. It was noted that the organic phase dispersed more rapidly into the aqueous phase, resulting in smaller LNPs, thus suggesting that faster mixing correlates with reduced nanoparticle size.^34,51^ Consequently, microfluidic designs that quickly achieved a 90% mixing index were deemed to be more effective.

**Figure 2:**
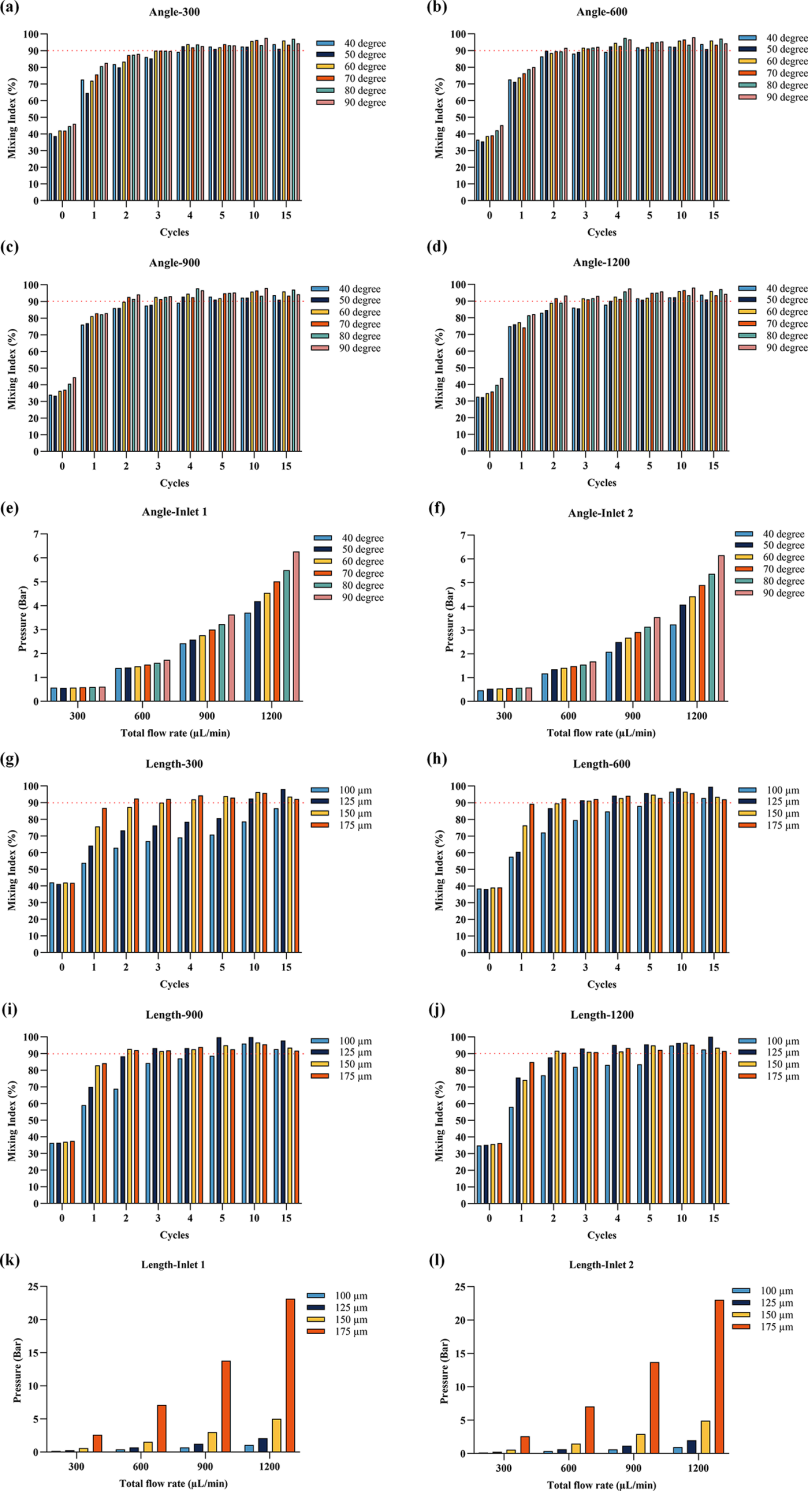
Mixing index and pressure drop results for different structural designs: Mixing indexes for various baffle angles (a) at a total flow rate of 300 μL/min; (b) at a total flow rate of 600 μL/min; (c) at a total flow rate of 900 μL/min; and (d) at a total flow rate of 1200 μL/min. (e) Pressure drop at Inlet 1 for different baffle angles. (f) Pressure drop at Inlet 2 for different baffle angles. Mixing indexes for various baffle lengths with a fixed 70° angle (g) at a total flow rate of 300 μL/min; (h) at a total flow rate of 600 μL/min; (i) at a total flow rate of 900 μL/min; and (j) at a total flow rate of 1200 μL/min. (k) Pressure drop at Inlet 1 for different baffle lengths with a fixed 70° angle. (l) Pressure drop at Inlet 2 for different baffle lengths with a fixed 70° angle.

The mixing indexes for different baffle angles are presented in Figure 2a–2d. At a low flow rate of 300 μL/min (Figure 2a), the 50° baffle angle exhibited the smallest mixing index at 38.7%, while the 90° baffle angle had the largest with 46%. Baffle angles larger than 50° were the first to reach a 90% mixing index after four cycles, whereas 40° required five cycles. At an increased total flow rate of 600 μL/min (Figure 2b), baffle structures with 60, 70, 80, and 90° angles reached a 90% mixing index after three cycles. At 900 μL/min (Figure 2c), structures with 60, 70, 80, and 90° angles achieved a 90% mixing index after three cycles. At a total flow rate of 1200 μL/min (Figure 2d), the 70 and 90° baffle structures exceeded a 90% mixing index after two cycles. The pressure drop simulation results are displayed in Figure 2e,2f, showing that both the flow rate and baffle angle increase the pressure drop at inlets 1 and 2. Given that cyclic olefin copolymer (COC) chips will be used in future production and air pressure will be employed to drive the mixing of the solutions, both mixing performance and pressure drop must be considered when selecting the optimal structure, while maintaining proper achievable pressure from the pressure pump and bonding strength of the chip. The control board has been ordered with a maximum pressure rating of 7 bar. The 70° baffle structure, which reached a 90% mixing index early and had a lower pressure drop compared to those of the 80 and 90° structures, was selected to study the effect of baffle length on mixing performance and pressure drop.

The mixing indices for different baffle lengths are shown in Figure 2g–2j. At a low total flow rate of 300 μL/min (Figure 2g), the mixing index increased with the baffle length. The 175 μm structure was the first to reach a 90% mixing index after two cycles and maintained this after four cycles. After 15 cycles, the 100 μm baffle structure had a mixing index of 86.6%, which was unsatisfactory. At a total flow rate of 600 μL/min, the structures with 125 and 150 μm lengths performed better, both reaching a 91% mixing index after three cycles. When the total flow rate was 900 and 1200 μL/min, the mixing indices of structures with 150 and 175 μm lengths were similar, with the 100 and 125 μm structure still performing poorly. The pressure drops, depicted in Figure 2k,2l, increased with both total flow rate and baffle length, particularly from 150 to 175 μm, where the pressure drop escalated sharply.

### 3.2 Physical Properties of Empty LNPs

Based on previous simulation results, PDMS microfluidic chips were used to investigate how baffle structure, total flow rate, and dilution affect lipid nanoparticle size and PDI. PDMS chips were manufactured using UV lithography on silicon wafers with each chip used only once. Lipids were dissolved in ethanol at a concentration of 8 mg/mL to serve as the organic phase. The weight ratio of DDAB:cholesterol:DSPC:DMG-PEG 2000 was 40:48:10:2. A 10 mM Tris buffer was used as the aqueous phase. The organic phase was transferred to a syringe connected to inlet 2, while the aqueous phase was transferred to another syringe connected to inlet 1. Two syringe pumps were used to push the two phases, with the flow rate set according to the total flow rate (300, 600, 900, and 1200 μL/min) and a flow rate ratio of aqueous phase to organic phase of 3:1.

The size and PDI of the LNPs are shown in Figure 3. For structures with different baffle angles, the size of the LNPs diluted by Tris buffer or PBS buffer was smaller than that without any dilution (Figure 3a–d). At a low total flow rate of 300 μL/min (Figure 3a), increasing the baffle angle slightly reduced the size of the LNPs until the structure with a 70° baffle reached 164.67 nm, after which the size increased. After dilution with Tris buffer and PBS buffer, LNPs with a 70° baffle had smaller sizes of 98.59 and 103.61 nm, respectively. The PDI values of all groups were lower than 0.2.

**Figure 3:**
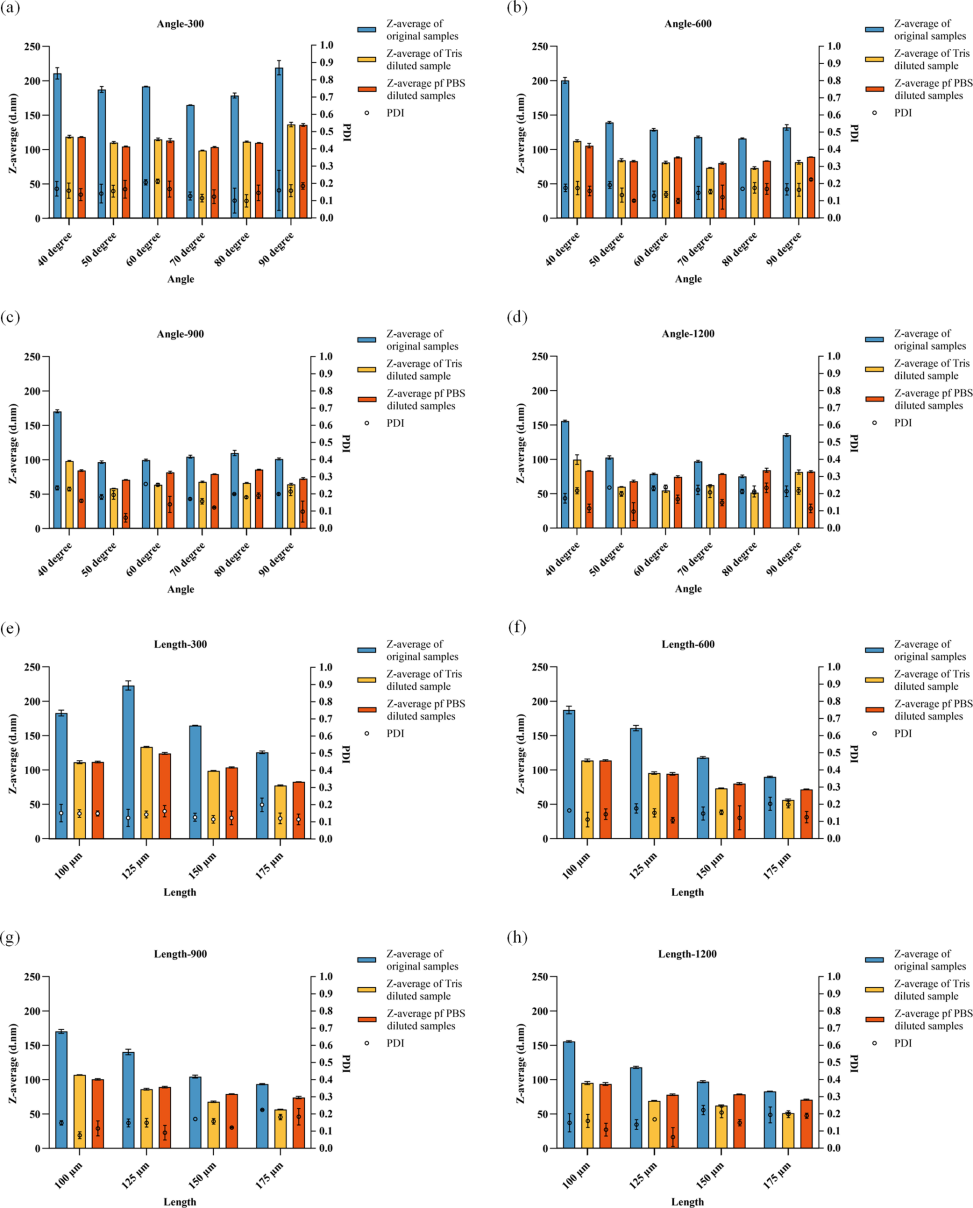
Lipid nanoparticle size and PDI results for different structural designs: Size and PDI results for various baffle angles (a) at a total flow rate of 300 μL/min; (b) at a total flow rate of 600 μL/min; (c) at a total flow rate of 900 μL/min; and (d) at a total flow rate of 1200 μL/min. Size and PDI results for various baffle lengths (e) at a total flow rate of 300 μL/min; (f) at a total flow rate of 600 μL/min; (g) at a total flow rate of 900 μL/min; and (h) at a total flow rate of 1200 μL/min.

As the flow rate increased to 600 μL/min (Figure 3b), the sizes of all LNPs decreased. The 80° structure produced the smallest LNPs at 115.99 nm, with the 70° structure yielding a size of 118.12 nm. The diluted LNPs showed similar results, with the 70° and 80° structures having the smallest sizes. The PDI of all groups remained below 0.2. At 900 μL/min (Figure 3c), the sizes of the LNPs, whether undiluted or diluted with Tris buffer or PBS buffer, differed slightly from the previous results. The 50° structure produced smaller LNPs, measuring 96.50, 58.33, and 70.72 nm, respectively. At this flow rate, the PDI increased, with some groups exceeding 0.2. The 70° structure had the smallest PDI values of 0.171, 0.157, and 0.121. As the flow rate continued to increase to 1200 μL/min (Figure 3d), the size further decreased, with the 80° structure producing the smallest nanoparticles at 75.23, 51.51, and 84.16 nm. The PDI of all groups was around 0.2.

Based on previous simulation results and the physical properties of empty LNPs, the 70° structure was selected to study the effect of the baffle length on the physical properties of LNPs. The size and PDI results for structures with baffle lengths of 100, 125, 150, and 175 μm at different total flow rates are shown in Figure 3e–3h. These figures indicate that the sizes of LNPs diluted by Tris buffer and PBS buffer were consistently smaller than those without dilution. At different total flow rates, increasing the baffle length resulted in smaller LNPs. Additionally, increasing the total flow rate led to a decrease in the nanoparticle size. The PDI values of most groups remained below 0.2, except for the 175 μm baffle length, which had a higher PDI.

Overall, the 70° structure demonstrated better size and PDI results. Although the 175 μm baffle length produced smaller LNPs, its PDI was suboptimal. Additionally, simulation results indicated that the 175 μm structure had a larger pressure drop. Therefore, the 70° structure with a 150 μm baffle length was chosen for preparing LNPs with GFP mRNA and testing their transfection efficiency.

### 3.3 Preparation of LNPs Encapsulating GFP mRNA

To test the functionality of LNPs *in vitro*, PDMS chips with a 70° angle and 150 μm baffles were used to prepare LNPs carrying GFP mRNA. The preparation process is illustrated in Figure 4a. Lipids were dissolved in ethanol to create the organic phase,^52^ using the same formulation as previously described. GFP mRNA was diluted in Tris buffer to concentrations of 0.04, 0.08, and 0.16 mg/mL to serve as the aqueous phase. The organic phase was transferred to a syringe connected to inlet 2, while the aqueous phase was transferred to another syringe connected to inlet 1. The organic phase was flowed at 300 μL/min, and the aqueous phase was flowed at 900 μL/min. LNPs were collected from the outlet, approximately 1 mL, and diluted five times with PBS buffer.

**Figure 4:**
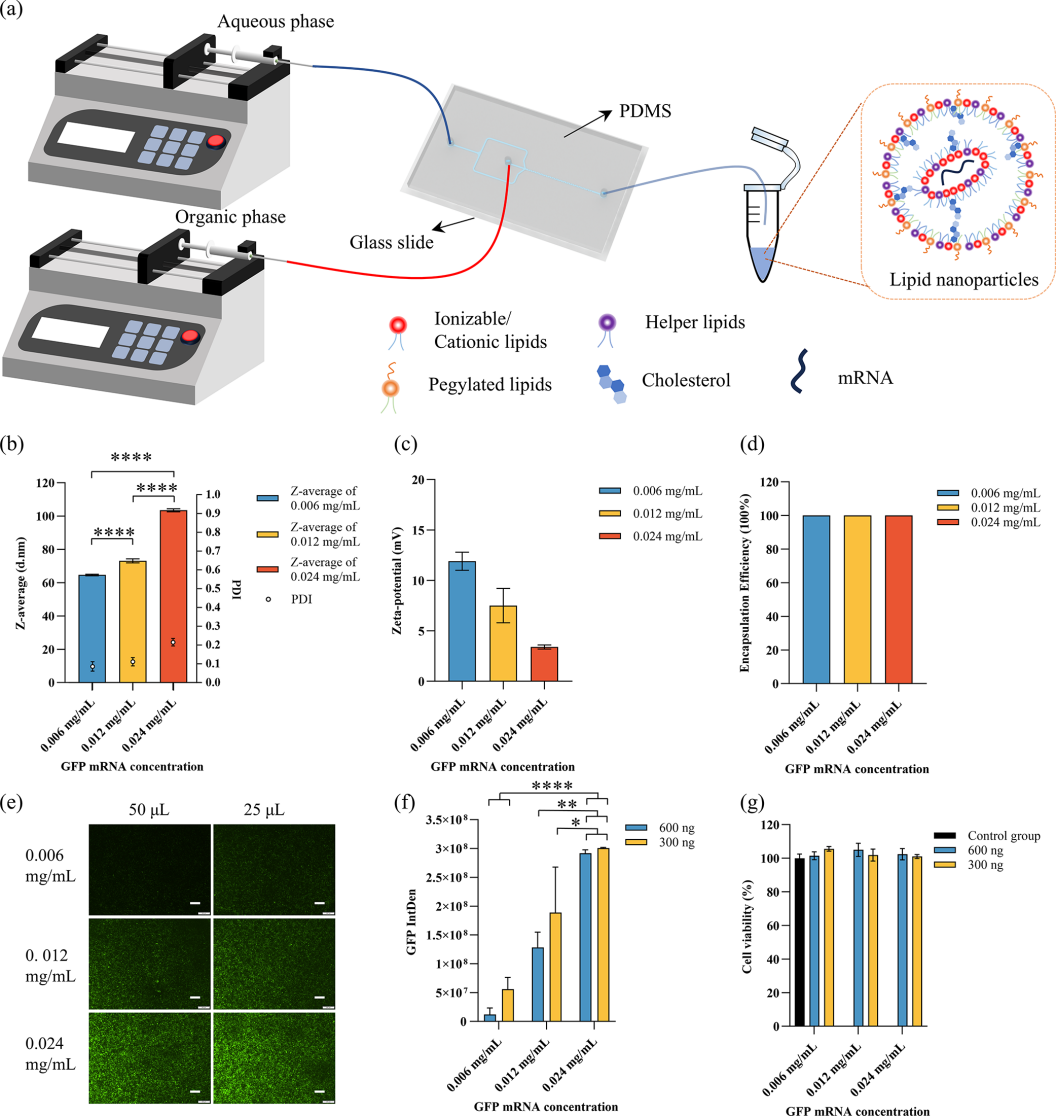
LNPs with GFP mRNA and cell experiments: (a) Preparation process; (b) size and PDI results of LNPs with different GFP mRNA concentrations; (c) zeta potential results of LNPs with different GFP mRNA concentrations; (d) encapsulation efficiency results of LNPs with different GFP mRNA concentrations; (e) fluorescence microscopy images of GFP expression captured with a 4× objective. Scale bars represent 200 μm. (f) Integrated fluorescence intensity related to GFP expression, semiquantified using ImageJ software. (g) Cell viability of HEK cells tested using the alamarBlue assay.

The size and PDI of the LNPs with different GFP mRNA concentrations are shown in Figure 4b. As the GFP mRNA concentration increased, both the size and the PDI of the nanoparticles also increased. The sizes were 64.63, 73.09, and 103.46 nm for the different concentrations, while the PDIs were 0.086, 0.111, and 0.215, respectively. The zeta potential results are shown in Figure 4c, with values of 11.9, 7.5, and 3.4 mV for the different GFP mRNA concentrations. The encapsulation efficiency was consistently higher than 98% across all concentrations, as shown in Figure 4d.

HEK cells were then used to test the transfection efficiency of the LNPs carrying GFP mRNA, with the results shown in Figure 4e–4g. Fluorescence images were taken after 48 h of culture and analyzed semiquantitatively using ImageJ. As the GFP mRNA concentration increased, GFP expression also increased. At lower GFP mRNA concentrations (0.006 and 0.0012 mg/mL), the group with 25 μL of GFP mRNA showed more GFP expression than the group with 50 μL. At higher concentrations, GFP expression was similar between the groups with 25 μL ng and 50 μL of GFP mRNA. Cell viability results indicated that all LNPs maintained good cell viability (Figure 4f).

## 4 Discussion

This study focused on the preparation of LNPs encapsulating mRNA with a small size and low PDI using PDMS microfluidic chips with specifically designed tilted rectangular baffle structures. Traditional methods of LNP preparation, such as pipet or vortex mixing, are time-consuming and often suffer from poor reproducibility. Although other researchers have used herringbone structures for LNP preparation,^40^ these typically involve complex two-layer designs, which can be challenging to manufacture via UV lithography for rapid optimization. Our work offers a novel approach by utilizing single-layer baffle structures with varying angles and lengths, simulated using CFD. The results demonstrated that these microfluidic chips not only are easy to manufacture but also offer excellent reproducibility and controllability, thus providing a more efficient method for LNP formulation screening and eventual clinical application.

Simulation results indicated that, as the two-phase solutions converge—consistent with previous findings—they begin to mix and spread.^49^ While several researchers have explored how rectangular baffle structures affect mixing performance,^53,54^ they typically employed Y-type structures to facilitate solution mixing. However, these Y-type designs exhibited a lower mixing index, compared to the flow focusing design used in our study. Additionally, they often used a 1:1 flow rate ratio, whereas a 3:1 ratio is typically employed for lipid nanoparticle preparation. Furthermore, previous studies did not simultaneously examine the effects of baffle angle and baffle length on both the mixing index and pressure drop and used those designs to prepare any nanoparticles.

In our study, when two-phase solutions passed through channels without baffles, the mixing index remained around 40%, even with increased total flow rates. This result was better than that observed with Y-type designs,^55,56^ likely due to insufficient mixing, which was driven primarily by tangential forces and a limited diffusion surface. However, once the solution passed through the baffle structure, mixing improved significantly, as the narrowing channels caused the solutions to flow more quickly and uniformly. Changes in the baffle angle altered the vortex formation behind the baffles, which in turn affected the mixing index. Smaller baffle angles allowed for a smoother flow, resulting in lower pressure drops. While the 70, 80, and 90° baffle angles produced similar mixing indices, the 70° angle exhibited the lowest pressure drop, making it the preferred choice. Additionally, the baffle length had a more pronounced impact on both mixing performance and pressure drop than the baffle angle. Longer baffles created smaller cross sections for solution passage, thereby enhancing the mixing. However, this also led to higher pressure drops. Notably, when the baffle length increased from 150 to 175 μm, the pressure drop increased substantially. Given that our control board had a maximum pressure limit of 7 bar, we identified the optimal parameters as a 70° baffle angle and a 150 μm baffle length based on these simulation results.

PDMS microfluidic chips were fabricated by using these designs and then used to prepare empty LNPs to study the effect of baffle structures. For LNPs without any dilution, increasing the total flow rate resulted in smaller nanoparticles due to faster mixing, consistent with simulation results. Carla et al.^57^ demonstrated a decrease in LNP size with increasing flow rate up to a certain threshold, beyond which further increases in flow rate had minimal effect on particle size and PDI. As the formation of LNPs is achieved by diluting ethanol in a buffer solution,^34^ once a certain flow rate is reached, the dilution factor of ethanol stabilizes, preventing further size reduction. At low flow rates, the 70° structure showed good size and PDI results. At high flow rates, no significant difference was observed between structures with baffle angles from 50 to 90°. The 70°, 150 μm structure was selected for further work. The ratio between the aqueous phase and the organic phase was 3:1, corresponding to an ethanol concentration of 25% in sodium acetate. The lipid nanoparticle structure is shown in Figure 4a.^58,59^ Lipids have a hydrophilic head and a hydrophobic tail, meaning that the exterior of lipid nanoparticles is water, while the interior contains ethanol. When Tris buffer or PBS buffer was used to dilute the lipid nanoparticles, the ethanol concentration decreased, prompting self-assembly into new structures. This is why lots of researchers use samples after dilution.^57,60^ Two buffers were used for dilution: Tris buffer resulted in smaller nanoparticles but larger PDI compared with PBS buffer, which was also used for washing cells, making it the final choice for dilution. Tris buffer was selected because Tris buffer was used to prepare lipid nanoparticles, and PBS was a general solution to dilute LNPs.^25^

DDAB, a cationic lipid, carries a positive charge, while mRNA carries a negative charge. When the organic and aqueous phases mix, the mRNA attracts many lipids through electrostatic interactions, particularly positively charged cationic lipids like DDAB, resulting in the formation of larger LNPs as surface energy decreases.^61^ Kulkarni et al.^62^ similarly found that formulations with a lower N/P ratio, containing more mRNA, led to increased size and PDI, which can influence the functionality of the LNPs, consistent with our findings. Specifically, more mRNA in these low N/P ratio formulations causes greater aggregation of cationic lipids near the mRNA, leading to a reduction in the zeta potential. In addition to the impact on particle size and charge, lipid nanoparticles used for nucleic acid delivery demonstrate the advantage of high encapsulation efficiency, with all groups achieving more than 98% efficiency. This encapsulation efficiency is critical for effective delivery, but the functional outcome also depends on optimizing the N/P ratio. Interestingly, Kulkarni et al.^62^ found that an N/P ratio of 6 provided optimal conditions for maximum gene expression, a finding corroborated by our experiments. We observed that decreasing the N/P ratio led to higher GFP protein expression, with an N/P ratio of 5.6 yielding similar results, indicating that higher mRNA content at these lower N/P ratios enhances the functional performance of the LNPs in terms of gene expression.

## 5 Conclusions

In this study, we explored structural design, simulation, and cell experimental verification for preparing LNPs. Initially, tilted rectangular baffle structures with various angles and lengths were designed and simulated. Subsequently, PDMS microfluidic chips were manufactured according to these designs and used to prepare the LNPs. The size and PDI results along with the simulation outcomes led to the selection of a structure with a 70° angle and 150 μm baffle length for preparing LNPs carrying GFP mRNA. Finally, different mRNA concentrations were tested, showing that higher mRNA concentrations resulted in higher transfection efficiency.

Having identified an optimal structure for LNP preparation, the next step is to develop a systematic microfluidic platform using plastic microfluidic chips. This platform will enable faster, more efficient, and highly reproducible LNP production, significantly accelerating the formulation process and facilitating the rapid clinical application of LNPs. It will also effectively tackle the challenge of quickly screening suitable formulations for clinical use.

## Supplementary Material



## Notes

Comparison of CFD simulation results for concentration profiles from top and different locations across the channel with different total flow rate and baffle angle (Figure S1); comparison of CFD simulation results for pressure drop profiles from top with different total flow rate and baffle angle (Figure S2); comparison of CFD simulation results for concentration profiles from top and different locations across the channel with different total flow rate and baffle length (Figure S3); and comparison of CFD simulation results for pressure drop profiles from top with different total flow rate and baffle length (Figure S4) (PDF)
