# Supplementary material for: Optimizing Microfluidic Channel Design with Tilted Rectangular Baffles for Enhanced mRNA-Lipid Nanoparticle Preparation
Source: ACS Biomater Sci Eng. 2025 May 21;11(6):3762–72. doi: 10.1021/acsbiomaterials.4c02373 (PMC12221266; doi:10.1021/acsbiomaterials.4c02373)
Supplement: Supplementary file 1 [file ab4c02373_si_001.pdf]

Supporting information for

Optimizing Microfluidic Channel Design with Tilted Rectangular Baffles for Enhanced mRNA-Lipid Nanoparticle Preparation

Mingzhi Yu[1], Dongsheng Liu[2,3], Pranay Shah[1], Bei Qiu[4], Allen Mathew[1], Liang Yao[4], Tianyu Guan[1], Hengji Cong[1], Nan Zhang[1*]

1. Centre of Micro/Nano Manufacturing Technology (MNMT-Dublin), School of Mechanical & Materials Engineering, University College Dublin, Dublin 4, Ireland

2. Department of Aerospace and Mechanical Engineering, South East Technological University, Carlow, Ireland.

3. The Centre for Research and Enterprise in Engineering (engCORE), South East Technological University, Carlow, Ireland

4. Charles Institute of Dermatology, School of Medicine, University College Dublin, D04 V1W8, Dublin, Ireland

[*] Corresponding author:

Nan Zhang (nan.zhang@ucd.ie)

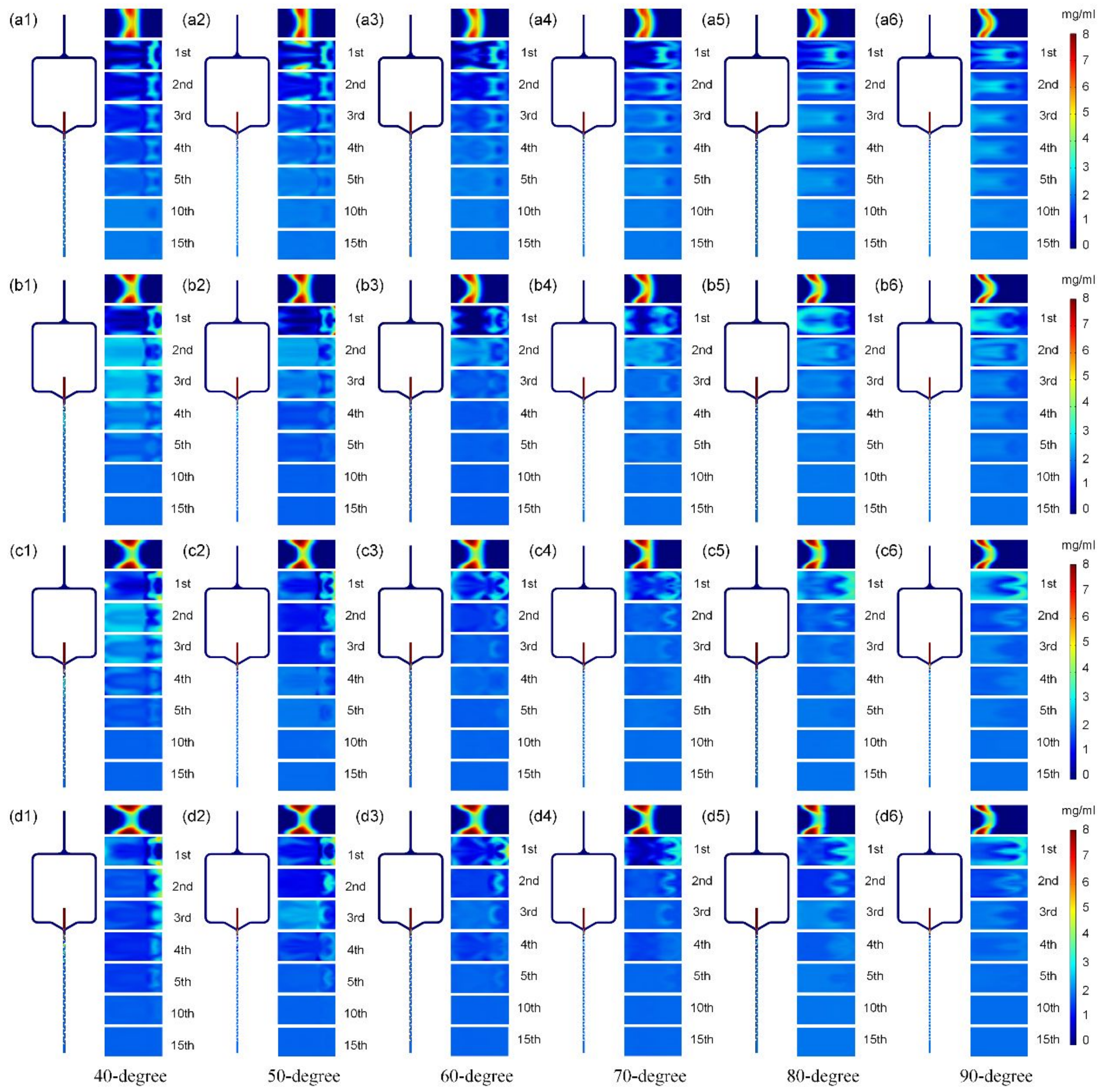

Figure S1. Comparison of CFD simulation results for concentration profiles from top and different locations across the channel with different total flow rate and baffle angle: (a) Different baffle angle for the flow rate of 300 μL/min; (b) Different baffle angle for the flow rate of 600 μL/min; (c) Different baffle angle for the flow rate of 900 μL/min; (d) Different baffle angle for the flow rate of 1200 μL/min.

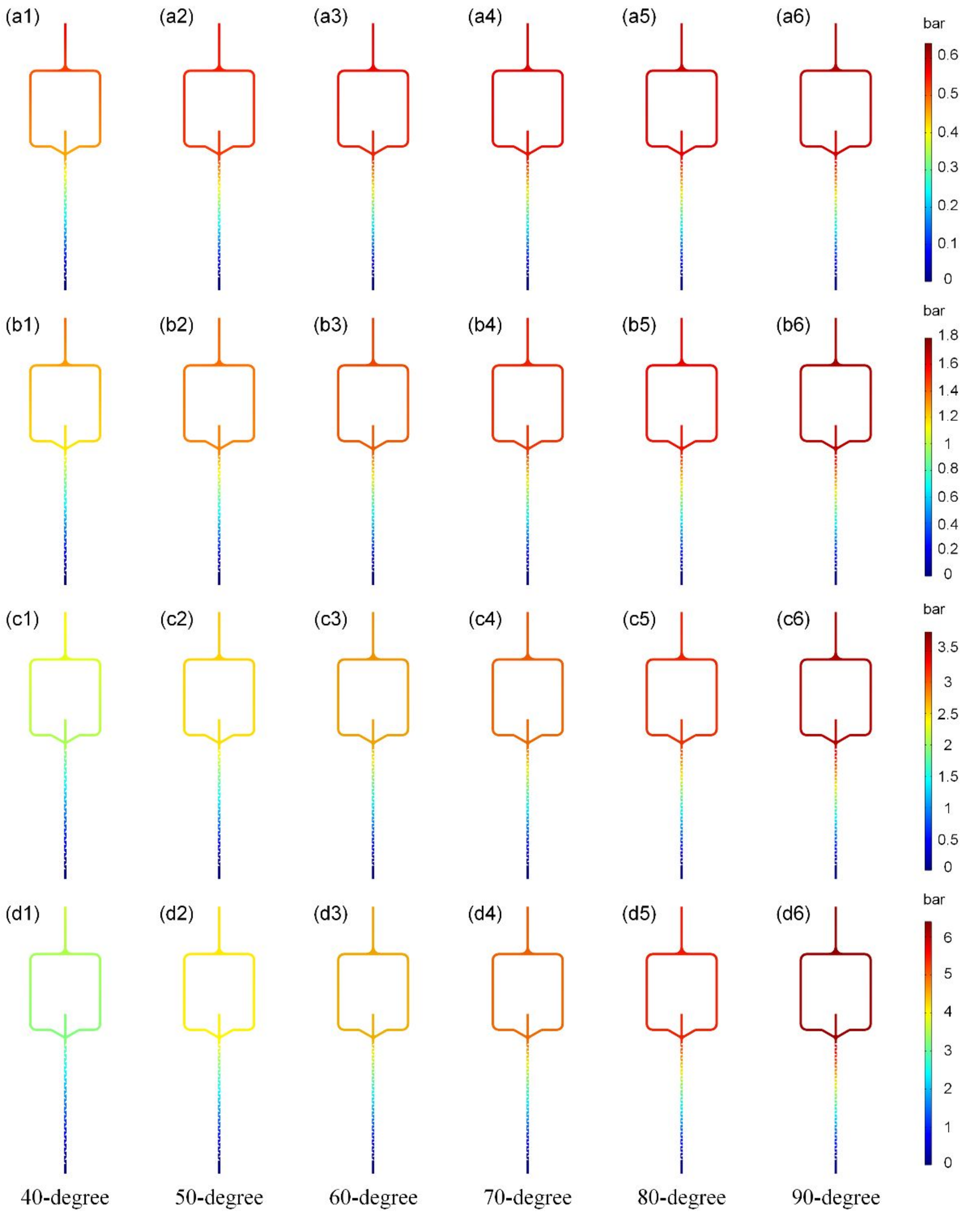

Figure S2. Comparison of CFD simulation results for pressure drop profiles from top with different total flow rate and baffle angle: (a) Different baffle angle for the flow rate of 300 μL/min; (b) Different baffle angle for the flow rate of 600 μL/min; (c) Different baffle angle for the flow rate of 900 μL/min; (d) Different baffle angle for the flow rate of 1200 μL/min.

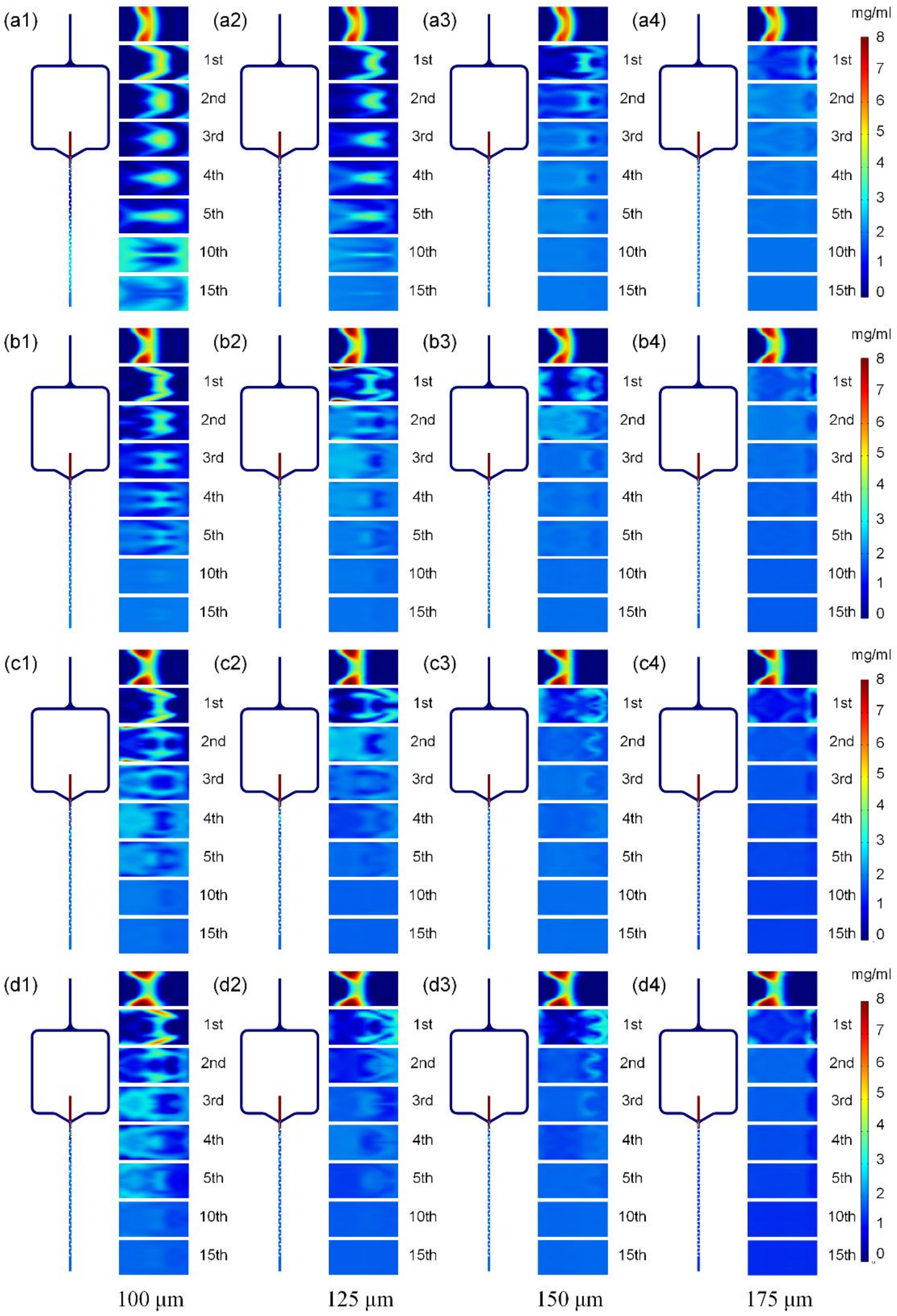

Figure S3. Comparison of CFD simulation results for concentration profiles from top and different locations across the channel with different total flow rate and baffle length: (a) Different baffle length for the flow rate of 300 μL/min; (b) Different baffle length for the flow rate of 600 μL/min; (c) Different baffle length for the flow rate of 900 μL/min; (d) Different baffle length for the flow rate of 1200 μL/min.

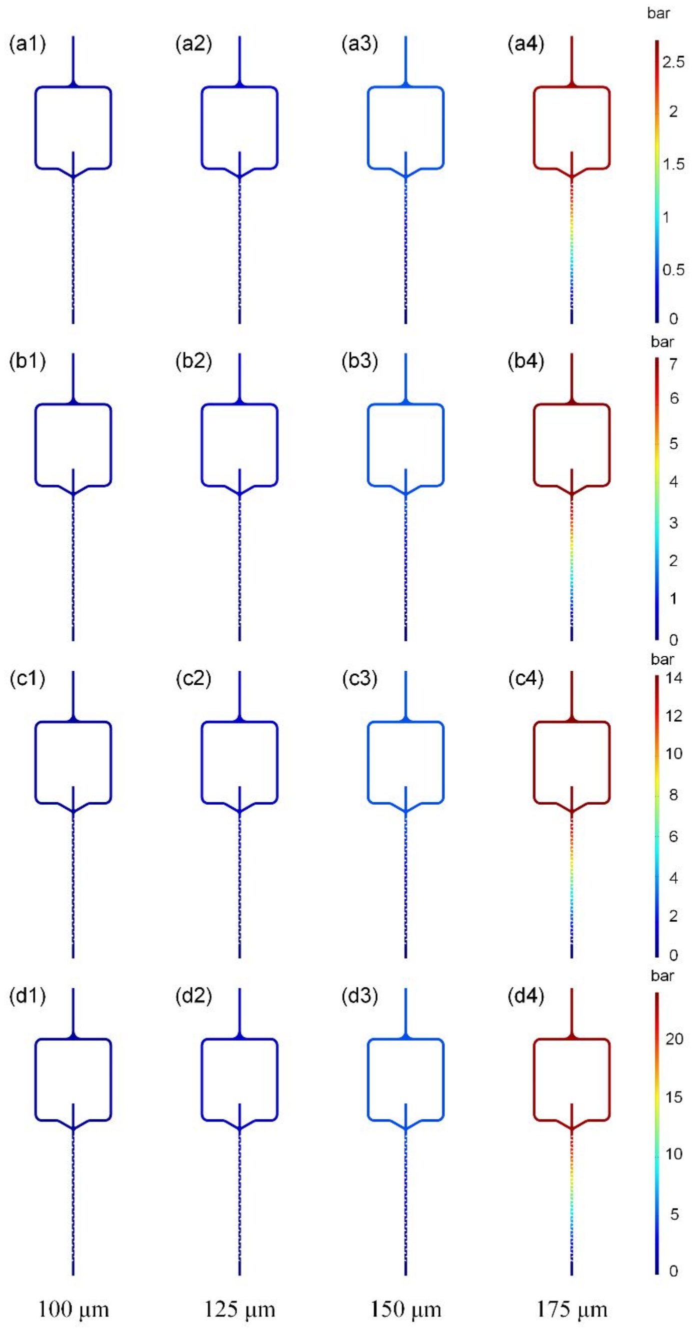

Figure S4. Comparison of CFD simulation results for pressure drop profiles from top with different total flow rate and baffle length: (a) Different baffle length for the flow rate of 300 μL/min; (b) Different baffle length for the flow rate of 600 μL/min; (c) Different baffle length for the flow rate of 900 μL/min; (d) Different baffle length for the flow rate of 1200 μL/min.
